# The Effect of Coconut Oil Addition to Feed of Pigs on Rectal Microbial Diversity and Bacterial Abundance

**DOI:** 10.3390/ani10101764

**Published:** 2020-09-29

**Authors:** Michal Rolinec, Juraj Medo, Michal Gábor, Martina Miluchová, Daniel Bíro, Milan Šimko, Miroslav Juráček, Ondrej Hanušovský, Zuzana Schubertová, Branislav Gálik

**Affiliations:** 1 Department of Animal Nutrition, Slovak University of Agriculture in Nitra, Trieda A. Hlinku 2, 94976 Nitra, Slovakia; daniel.biro@uniag.sk (D.B.); milan.simko@uniag.sk (M.Š.); miroslav.juracek@uniag.sk (M.J.); ondrej.hanusovsky@uniag.sk (O.H.); branislav.galik@uniag.sk (B.G.); 2 Department of Microbiology, Slovak University of Agriculture in Nitra, Trieda A. Hlinku 2, 94976 Nitra, Slovakia; juraj.medo@uniag.sk; 3 Department of Genetics and Animal Breeding Biology, Slovak University of Agriculture in Nitra, Trieda A. Hlinku 2, 94976 Nitra, Slovakia; michal.gabor@uniag.sk (M.G.); martina.miluchova@uniag.sk (M.M.); 4 Institute of Biodiversity Conservation and Biosafety, Slovak University of Agriculture in Nitra, Trieda A. Hlinku 2, 94976 Nitra, Slovakia; zuzana.schubertova@uniag.sk

**Keywords:** pig, coconut oil, lauric acid, rectal swab, intestinal microbiome

## Abstract

**Simple Summary:**

Looking for non-antibiotic substances that can enhance health by improving the gastrointestinal microbiome of animals is an ongoing task. Among other compounds, medium-chain fatty acids, such as lauric acid, can exert positive effects. Coconut oil is a rich source of lauric acid, and therefore, the aim of this study was to describe the effect of adding coconut oil to the feed of growing pigs on intestinal microbiome diversity and bacterial abundance. Rectal swab samples were analyzed to assess the intestinal microbiomes of pigs. Typically, growing pigs are characterized by continuously changing bacterial communities as a result of aging. However, a significant effect of coconut oil treatment was detected in the presented study. Decreases in *Corynebacterium*, *Pseudomonadales*, and *Mitsuokella* and increases in *Alloprevotella*, *Bifidobacteriales*, and *Lactobacilli* could be attributed to the supplementation of feed with coconut oil. Coconut oil treatment did not have a significant effect on the diversity index of rectal microbiomes, but an abundant increase in probiotics such as *Bifidobacterium* and *Lactobacillus* in the gastrointestinal tract is desirable in pig breeding. From this point of view, the addition of coconut oil to the feed of pigs is a good option for improving the microbiome in their gastrointestinal tracts.

**Abstract:**

Coconut oil has a high content of lauric acid, which has selective antibacterial activity. This study aimed to explore the effect of coconut oil ingestion on the gastrointestinal microbiomes of pigs. A 14-day-long feeding experiment included 19 pigs in two groups (9 on a normal diet and 10 on a diet supplemented with coconut oil). At the start and end of the experiment, a rectal swab sample was taken from each pig in both groups, and total bacterial DNA was extracted. We used 16S *r*RNA high-throughput amplicon sequencing to evaluate the microbiome changes during the feeding experiment. A total of 446 operational taxonomic units (OTUs) were detected in the whole sample set. Shannon’s indices of bacterial diversity did not change significantly during the experiment. Changes in the bacterial community during the study period and in response to the coconut oil treatment were highly significant (*p* < 0.001). During the study, an increase in the abundance of *Lactobacillus* was detected in the group treated with coconut oil. An increase in *Alloprevotella, Bifidobacteriales*, and *Lactobacillales* and a decrease in *Corynebacterium*, *Mitsuokella, Psychrobacter*, and *Pseudomonadales* were attributed to the coconut oil treatment. Although the addition of coconut oil to pig feed did not affect Shannon’s index of diversity, it had a positive effect on the abundance of bacterial groups that are considered to be commensal and/or probiotic.

## 1. Introduction

The gastrointestinal tract (GIT) is a multifactorial apparatus that assures the absorption of nutrients, water, and electrolytes and simultaneously protects the organism from pathogens and toxins [1]. Bacteria of the GIT play an essential role in disease prevention, the maintenance of the appropriate structure of the intestinal wall, and immune function [2]. According to Willing and Van Kessel [1], the microbial diversity of the intestine affects the functional development of the GIT in many aspects. Epithelial cells, together with specific proteins that are between these cells, provide a physical barrier against pathogens. The interaction of epithelial cells with the intestinal microbiome affects the rate of cell replacement and thus also the effectiveness of growth. One of the main functions of the intestinal microbiome is to provide energy to the intestinal epithelium, which is driven by the synthesis of short-chain fatty acids. Intestinal goblet cells produce mucin, which creates a thick layer that is impassable to pathogens and toxins [3]. Che et al. [4] confirmed this claim and also emphasized the role of *Lactobacillus* spp. in increasing mucin production in the pig intestine. It is clear that the loss of diversity in the intestinal microbiome is related to an increased chance of GIT diseases. According to Fouhse et al. [5], the loss of the gut microbial ecosystem diversity dramatically increases the risk of gastrointestinal diarrhea and is also linked to an increase in immune-mediated diseases. It is dangerous when pathogenic *Escherichia coli*, *Campylobacter*, or *Salmonella* are detected as present or increased in the intestinal contents or in fecal samples.

A weaned pig represents an excellent example of the very important role played by the gastrointestinal microbiome. Weaning is a key period in the life of piglets. It involves the separation of piglets from dams and also the transition from highly digestible milk to less digestible feed mixtures. During the weaning period, feed and water intakes also decrease, which results in structural and functional changes in the GIT, followed by the occurrence of diarrhea and increased mortality [6,7,8]. Disruption of the GIT microbiome happens mainly during a short period after the weaning of pigs. An exact definition of this status is difficult, but in general, it is an imbalance in the gut microbiome, which is manifested by a decrease in obligate anaerobes such as *Clostridia* and *Bacteroidia* and at the same time by an increase in facultative anaerobes such as species from *Enterobacteriaceae* [9].

The presence of short-chain fatty acids in the GIT of pigs seems to be beneficial for intestinal microbiota [10]. However, Hanczakowska [11] noted that medium-chain fatty acids also play an important role in pig nutrition and GIT function. The group of medium-chain fatty acids containing caproic (C6), caprylic (C8), capric (C10), and lauric (C12) acid can already be partly absorbed through the stomach mucosa. Their corresponding medium-chain triacylglycerols can be absorbed in their intact forms by intestinal epithelial enterocytes and then hydrolyzed by microsomal lipases. Thus, they are a readily available source of energy, and they are capable of improving the intestinal epithelial mucosal structure. They are also characterized by strong antibacterial activity due to their ability to penetrate the semi-permeable membranes of bacteria and damage their internal structures [11]. Hanczakowska [11] also stated that given these properties, they could be a good supplement for weaned piglet feed. They improve piglet performance and can be used as feed antibiotic replacements. In addition, Puyalto et al. [12] reported that lauric acid is the primary fatty acid of coconut oil, which is present at approximately 45–53% of the total fatty acid content. The effect of coconut oil can indeed be attributed to the properties of lauric acid. Among all saturated fatty acids, lauric acid has the strongest antimicrobial activity against Gram-positive bacteria, some viruses, and fungi. In an in vitro study, among all tested saturated fatty acids, lauric acid showed the strongest inhibitory effect against the following Gram-positive organisms: *Staphylococcus aureus*, *Staphylococcus epidermidis*, beta-hemolytic streptococci (group A and non-group A), group D *Streptococcus*, *Bacillus subtilis*, *Sarcina lutea*, *Micrococcus* spp., *Nocardia asteroides*, *Corynebacterium* spp., *Pneumococcus*, and *Candida albicans*. With respect to *Clostridium perfringens*, lauric acid showed the highest antimicrobial activity, followed by myristic, capric, oleic, and caprylic acid.

Thus, we hypothesized that coconut oil has a positive effect on the structure of intestinal microorganisms, which means increasing the diversity of the GIT microbiome while supporting probiotic microbes. Therefore, the aim of this study was to determine the diversity and composition of the microbiome in the rectums of pigs fed a diet supplemented with coconut oil.

## 2. Materials and Methods

A 14-day-long feeding experiment was realized at the Sheep and Pig Farm in Žirany (GPS 48°22′42.8″ N, 18°11′00.9″ E), which belongs to a university farm in Kolíňany (Slovak University of Agriculture in Nitra, Slovakia). In total, 19 pigs of the Large White breed were used to determine the effect of adding coconut oil to feed rations on the diversity and composition of the microbiome in the rectum. Pigs were 9 weeks old with an average live weight of 22.5 ± 3.03 kg. The group with coconut oil supplementation (*n* = 10) and the group on a normal diet (*n* = 9) were randomly divided into two separate pens prior to the experiment. Both pens had straw bedding with daily manure removal. The microclimate during the experiment met the requirements for housing pigs. Pigs had *ad libitum* access to feed and water during the experiment. The length of the feeder allows the simultaneous intake of feed by all pigs in the pen. Before the start of this experiment, all pigs were housed in one pen and fed the same commercial feed mixture as that used during the experiment. Animal care throughout the whole experiment was in accordance with Directive 2010/63/EU on the protection of animals used for scientific purposes.

### 2.1. Experimental Diet and Animal Feeding

Pigs in the normal group were fed a commercial feed mixture (Afeed, Hustopeče, Czech Republic). Pigs in the oil group ingested feed rations that contained 99.7% commercial feed mixture and 0.3% commercial coconut oil for the duration of the experiment. The amount of coconut oil used represents a dose of 3 g/kg feed mixture, which was assessed according to previously published information about the addition of distilled coconut fatty acids (3 g/kg of pig feed) [12]. This dose is feasible for use in the commercial breeding of pigs. The feed ration composition, nutritional parameters, and the proportion of fatty acids in feed rations provided during the experiment are shown in Table 1.

### 2.2. Nutritional Parameter Determination of Feed Rations

Nutritional parameters and the proportion of fatty acids in feed rations provided during the experiment were determined at the Laboratory of Quality and Nutritive Value of Feeds at the Department of Animal Nutrition (Slovak University of Agriculture in Nitra, Slovakia) according to standard methods [13]. In brief, dry matter (DM) was determined gravimetrically after drying the sample at (103 ± 2 °C). Crude protein (CP) was evaluated as the total nitrogen content, as determined by the Kjeldahl method (Nx6.25). The lysine concentration was determined using an AAA 400 amino acid analyzer (Ingos, Prague, Czech Republic). The samples used for lysine determination were adjusted using acidic and oxidative acidic hydrolysis. Chromatographic analysis of sample hydrolysates was performed using Na-citrate buffers and ninhydrin detection according to Davídek et al. [14]. Crude fat (CFa) was determined by extraction and the gravimetric method according to the Soxhlet principle without previous acid treatment. Crude fiber (CFi) content was determined gravimetrically as the difference between residues after hydrolysis and after combustion. Ash (A) was determined by measuring the resulting inorganic residue weight after ignition in a Muffle furnace at 550 °C. Nitrogen-free extract (NFE) was calculated according to the formula NFE = DM − (CP + CFa + CFi + A). Starch was determined by the polarimetric method, and sugar was measured according to the Luff–Schoorl method. The concentration of metabolizable energy for pigs (MEpigs) was calculated according to the formula published in the Regulation of the Government of the Slovak Republic 440/2006 on feed mixtures. The fatty acid composition of feeds was analyzed in a manner similar to that in our previous study [15]. Briefly, the triglycerides were hydrolyzed (saponified) into glycerol and free fatty acids, which were derivatized to their methyl esters (FAMEs). After the FAMEs were prepared, they were separated on a DB-23 analytical column by gas chromatography (GC) with a flame ionization detector (FID). The analyses were performed on an Agilent 6890A GC analyzer (Agilent Technologies, Santa Clara, CA, USA). As the column reference, a 37-component mixture (Supelco 47885-U) was used.

### 2.3. Sample Collection

Rectal swab sampling was used because it allowed us to compare changes in the microbiome over time within a single individual pig. The microbiome composition profiles provided by rectal swabs are similar to those obtained from fecal samples, according to Choudhury et al. [16]. Three rectal swab samples (technical replicates) were taken from each pig before the treatment and also after 14 days (after the end of treatment). Rectal swab samples were taken with a sterile cotton swab (Heinz Herenz, Hamburg, Germany) that was inserted 40–50 mm into the rectum and rotated against the bowel wall. Immediately after sampling, swabs were placed and thoroughly mixed in 2 mL Eppendorf tubes prefilled with 600 µL of preservation reagents (DNA/RNA Shield^TM^; Zymoresearch, Irvine, CA, USA). The Eppendorf tubes with rectal swab samples were transported on ice to the laboratory, where they were stored at −80 °C.

### 2.4. Bacterial DNA Extraction

The total DNA was extracted from each collected rectal swab using the QIAamp DNA Stool Mini Kit (QIAgen, Germantown, MD, USA). Before extraction, swabs were washed in 1.4 mL of ASL lysis buffer. Then, a mechanical lysis step using the Precellys 24 homogenizer (Bertin Instruments, Montigny-le-Bretonneux, France) at 6800× *g* for 15 s was included in the protocol [17] before heating the suspension for 5 min at 95 °C. Further steps were performed according to the manufacturer’s instructions.

### 2.5. 16S rRNA Amplicon Sequencing

Hypervariable region V4 of the 16S rRNA gene was used for high-throughput sequencing analysis of pigs’ rectal microbiomes. Amplification of the selected region was carried out with a combination of primers 515F (5′-GTGCCAGCMGCCGCGGTAA-3′) and 806R (5′-GGACTACHVGGGTWTCTAAT-3′), which were evaluated by Caporaso et al. [18] for use with the Illumina MiSeq sequencer. Primers were enhanced by 6-base-long barcodes for sample identification. Reactions were carried out in a C1000 thermal cycler (Biorad, Hercules, CA, USA). The total volume of the reaction mixture was 15 µL, which consisted of 1 µL of the DNA sample, 1.5 µL of primer 515F with a concentration of 5 pmol.µL^−1^, 1.5 µL of primer 806R with a concentration of 5 pmol.µL^−1^, 7.5 µL of Q5 HS Mastermix (New England Biolabs, Ipswich, MA, USA), and 3.5 µL of MilliQ water. PCR consisted of the following thermal cycles: activation at 98 °C for 30 s, followed by 35 cycles (denaturation at 98 °C for 5 s, annealing at 63 °C for 15 s, and polymerization at 72 °C for 2 min), and final polymerization at 72 °C for 2 min.

PCR products were purified by the Jena PCR purification kit (Jena bioscience, Jena, Germany), quantified using Qubit (Thermo Fisher Scientific, Waltham, MA, USA), and pooled together. The Nextera XT indexing kit (Illumina, San Diego, CA, USA) was used for sequencing library preparation and adapter attachment. The library was quantified by qPCR and sequenced on an Illumina Miseq using V3 kit 2 × 300 bp.

### 2.6. DNA Sequencing Data Analysis

The sequence editor SEED ver. 2.1 [19] was used to process the acquired sequences. Forward and backward sequencing reads were joined using the FastQJoin software. Samples were identified according to their barcodes, which were subsequently removed. All three technical replicates were pooled into a single sample. Sequences with quality lower than Q30, as well as sequences shorter than 250 or longer than 350 bases, were excluded from further analysis. Chimera sequences were detected using Vsearch [20] and were also removed. The remaining sequences were clustered into operational taxonomic units (OTUs) using Vsearch software with a sequence identity threshold at 97%. The most abundant sequence was found for each OTU and identified using RDPClassifier [21] by comparison to the database SILVA NR_123. OTUs with an abundance of less than three, i.e., singletons and doubletons, were excluded from further analysis. The number of sequences was corrected according to the 16 rRNA gene copy numbers using rrnDB [22].

### 2.7. Statistical Analysis

The determined OTUs were processed as a contingency table in MS Excel (MS Office 2013). A Venn diagram was constructed using the venn package in the R environment [23]. The Shannon index of diversity was calculated in the vegan package [24] and compared using analysis of variance (ANOVA) (package aov) in R. Non-metric multidimensional scaling (NMDS) was used for visualization of sample dissimilarity with respect to the whole microbial community. Unifrac distances [25] based on the distribution of OTUs in samples, as well as the genetic similarity of OTUs acquired from PhyML maximum likelihood tree [26], were used for this analysis. Unifrac distances were also used for the comparison of microbiomes using permutational multivariate analysis of variance (PERMANOVA) in vegan. Relative abundances of microbiome members on different taxonomic levels between the control and experimental groups were evaluated by the Mann–Whitney U-test, while the effect of treatment was evaluated by the Wilcoxon Signed-Ranks test. For the 35 most common genera, relative differences ((after − before)/(after + before)) caused by treatments for all pigs were drawn using the Heatmap3 package [27] in R.

## 3. Results

In total, 1,832,416 raw sequence reads were acquired for all sequenced samples (technical replicates). Quality filtration, length trimming, and chimera removal resulted in 1,215,613 sequences, representing an average of 10,663 sequences per single technical replicate, i.e., 31,989 sequences for each pig. Sequences were deposited in GenBank under BioProject no. PRJNA637986.

**Figure 1:**
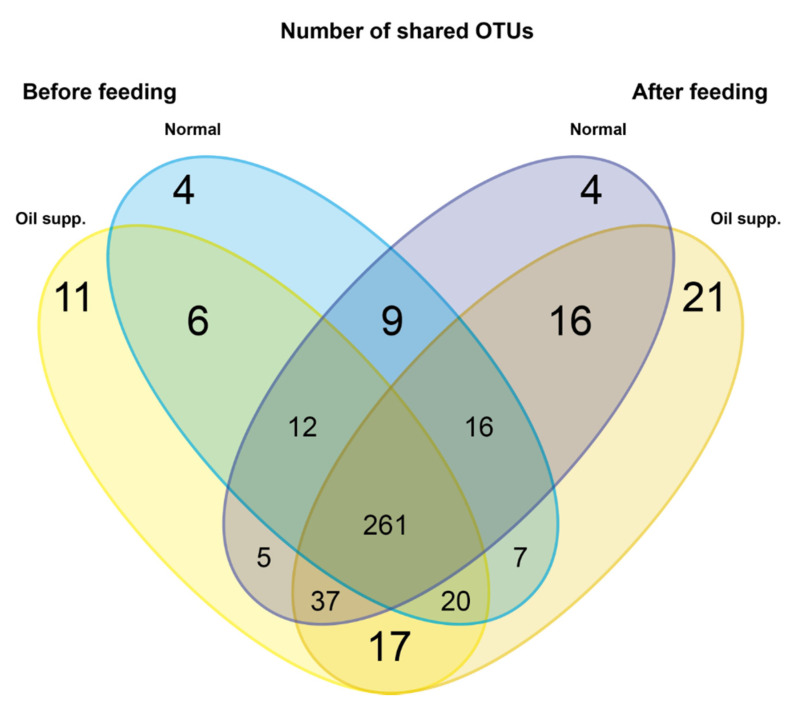
Venn diagram of operational taxonomic units (OTUs) shared between experimental and control groups of pigs before and after they were fed a normal diet with and without supplementation by coconut oil.

Clustering resulted in 446 OTUs, the majority of which was shared by all evaluated pigs (Figure 1). Most of the unique sequences (21) were found in the experimental group after treatment. The highest counts of two groups that share OTUs were found within the oil supplemented group (17) and in both treatments after the experiment (16).

**Figure 2:**
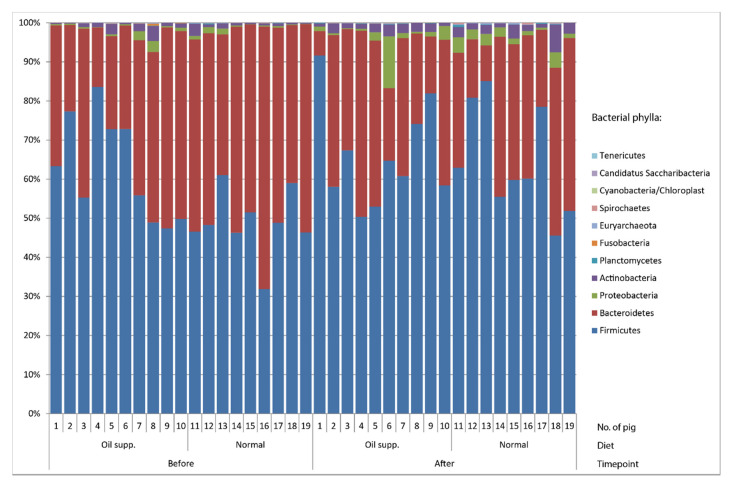
Bacterial phyla in the rectal swab of pigs fed a normal diet and a diet supplemented with coconut oil.

Regardless of treatment or sampling time point, *Firmicutes* and *Bacteroidetes* were the most abundant bacterial phyla in all groups of pigs (Figure 2). Other bacterial phyla represented less than 10% of the community in all samples except for two. A detailed structure of community members with occurrences of more than 0.5% is listed in Table 2. The genus *Megasphera* was predominant in three out of four sample groups. Together with other *Negativicutes* genera, *Dialister*, *Mitsuokella, Propionispira,* and *Selenomonas* represented the majority of *Firmicutes*, while other members of this phylum, such as the orders *Clostridialles* and *Lactobacillales*, were much less common, with only a 1–3% share. The genus *Prevotella* was predominant within *Bacteroidetes.* Diversity within this genus was considerably high, with a total of 89 OTUs, of which 21 had occurrences higher than 0.5% in at least one of the groups of pigs.

**Figure 3:**
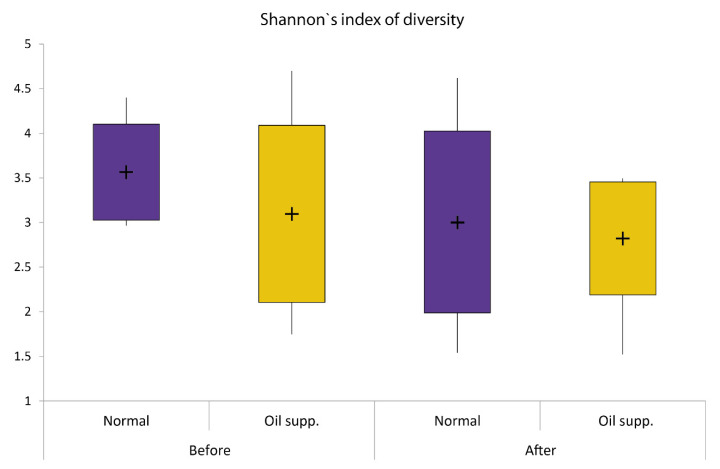
Shannon’s indices of diversity for experimental and control groups of pigs before and after they were fed a normal diet with and without supplementation with coconut oil. Boxes show interquartile range, whiskers defining min. and max. values, + signs are average values.

**Figure 4:**
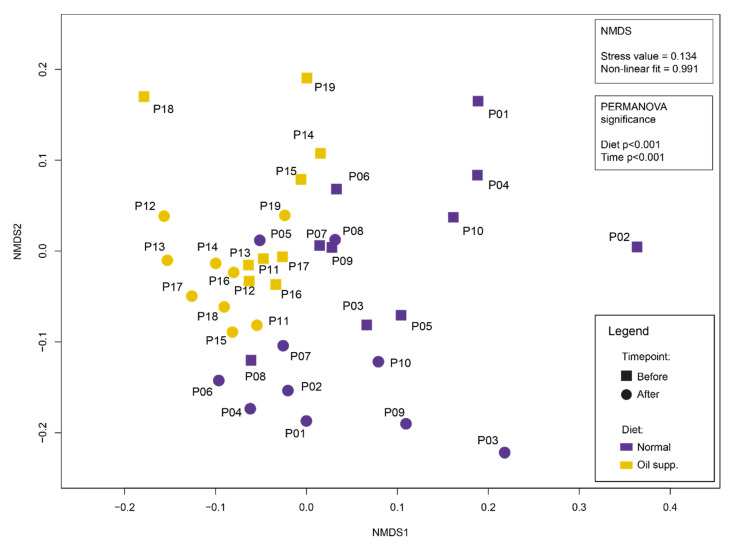
Non-metric multidimensional scaling (NMDS) plot of microbial community composition in the rectal swabs of pigs fed a normal diet and a diet supplemented with coconut oil.

Bacterial diversity expressed by Shannon’s index was not significantly different between groups either before or after treatment (ANOVA; F = 5.47; DF = 4; *p* = 0.458), but the variance within some groups was considerably high (Figure 3). However, NMDS analysis of unweighted Unifrac distances (Figure 4) provided detailed insight into changes in the bacterial community in pigs’ rectums. The microbiota communities in pigs were not very similar before treatment and changed during treatment. Moreover, changes that occurred during the feeding experiment were not the same between groups. Both factors, i.e., time and treatment, as analyzed by PERMANOVA, were highly significant.

Although the communities were not the same in the control and experimental groups before treatment, the number of significantly different OTUs increased from 29 to 64 after treatment when all generated OTUs were analyzed. The number of significant changes over time was similar for both groups. In the group fed by a commercial diet, 6 orders, 16 genera, and 39 OTUs significantly changed during the experiment, while in the oil-supplemented group, 6 orders, 20 genera, and 40 OTUs significantly changed. Significant changes in *Coriobacteriales* (increase), *Porphyromonadaceae* (increase), and *Selenomonas* (decrease) appeared in both treatments, and they can be attributed to the life stage. On the other hand, changes in *Corynebacterium* (decrease), *Alloprevotella* (increase), *Mitsuokella* (decrease), *Pseudomonadales* (decrease), *Bifidobacteriales* (increase), and certain lactobacilli (increase) can potentially be attributed to the supplementation of feed with coconut oil because their changes were opposite or very subtle in comparison with changes in the control group (Table 2.).

**Figure 5:**
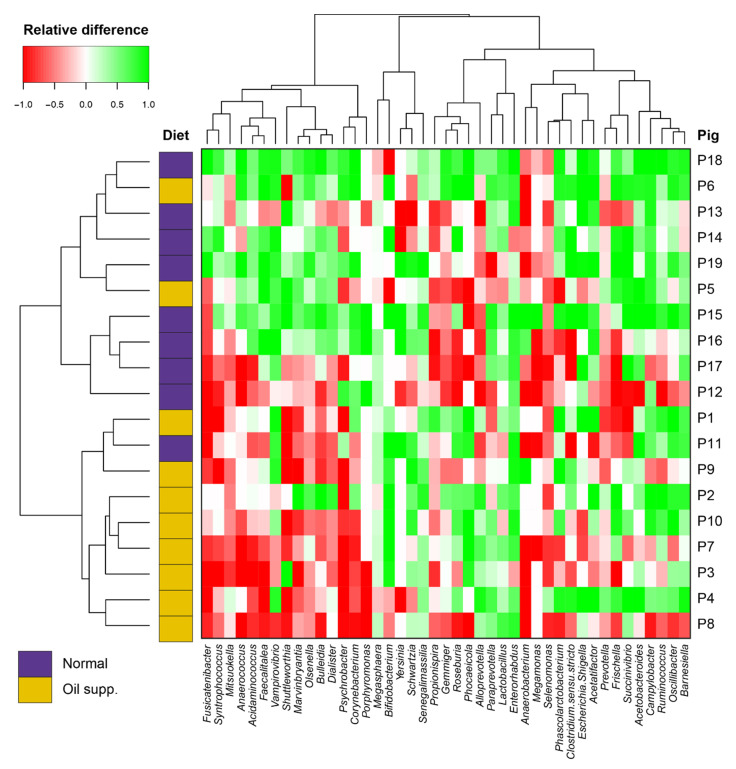
Heatmap of relative differences ((after − before)/(after + before); both 0 = 0) of the 35 most common genera in the rectal swabs of pigs fed a normal diet and a diet supplemented with coconut oil.

For a better illustration of changes in the microbial community, the relative differences in the 35 most common genera are plotted in a heatmap (Figure 5). In this heatmap, a value of −1 represents the disappearance of a particular genus, while a +1 value indicates that it rose from the initial value of 0. There is visible clustering of pigs according to diet. The abundance of genera from the first cluster (*Fusicatenibacter, Mitsuokella, Corynebacterium*, and *Porphyromonas*) increased mainly in the group fed the pure commercial diet, while it tended to decrease when coconut oil was added to the feed.

## 4. Discussion

The most abundant phyla found in the rectal microbiomes of pigs were *Firmicutes* and *Bacteroidetes*, followed by *Proteobacteria* and *Actinobacteria*. These results are in accordance with Choudhury et al. [16], who found the same order of abundance of these phyla in the rectal swabs of young pigs. According to Holman et al. [28], some bacterial groups in pig GITs represent a core microbiome and are not affected by sampling place, diet, age, or breed. Their analysis was based on 16S *r*RNA sequences from 20 studies of pig intestinal microbiomes, and 85% of the total sequences within the whole intestinal tract were represented by the phyla *Firmicutes* and *Bacteroidetes*, while *Prevotella*, *Clostridium*, *Alloprevotella*, *Ruminococcus*, and *RC9* groups were detected in 99% of feces samples. The observed diversity evaluated by Shannon’s index was in the typical range reported for rectal microbiomes in many studies [28].

Richards et al. [29] claimed that the intestinal microbiome of pigs is an ecosystem that develops dynamically, particularly in young pigs. During the two-week experiment, we observed an increase in *Firmicutes* and a decrease in *Bacteroidetes,* similar to the study of Choudhury et al. [16].

We encountered a rise in *Megasphaera* and a decrease in *Prevotella* in the microbiomes of pigs in both groups. This shift from *Prevotella* to *Megasphaera* did not have a negative effect on the health of the animals, as reported by Liao and Nyachoti [30]. Bajagai et al. [31] consider *Prevotella bryantii* and *Megasphaera elsdenii* to be probiotics commonly used in animal nutrition.

During the experiment, an increase in *Bifidobacteriales*, *Lactobacillus*, and *Megasphaera* together with a decrease in *Prevotella* was detected in both groups of pigs. Gresse et al. [32] reported that GITs with disrupted microbiomes are characteristic of a loss of bacterial diversity and decrease in *Lactobacillus* together with an increase in *Clostridium*, *Prevotella*, *Proteobacteria*, and *E. coli*. As we mainly observed the opposite changes in our study, we suppose that the pigs successfully adapted their microbiome to changed feeding conditions. The genus *Prevotella*, which had the second-highest abundance in this study, is connected to ingested saccharides in human nutrition [33,34]. Members of *Clostridiales*, such as *Clostridium*, *Blautia,* and *Ruminococcus*, which are similar to *Prevotella* and very common in the mammalian gastrointestinal tract [35], were much less abundant in our study. These genera produce short-chain fatty acids, mainly acetic acid, and therefore, *Prevotella* is able to produce energy sources for butyrate-producing bacteria [36,37]. Butyrate alleviates intestinal inflammation, and epithelial cells can use it as an energy source [38].

A positive trend in *Lactobacillus* abundance was determined for both groups. However, it increased significantly only in the group fed a diet supplemented with coconut oil. Bacteria from this genus have been reported to have a favorable effect on the intestinal tract [39]. The increase in *Lactobacillus* in the group that was fed coconut oil can be explained by the findings of Puyalto et al. [12], who concluded that coconut fatty acid intake could decrease the abundance of *Enterobacteriaceae* and increase that of *Lactobacillus* in the GIT of pigs at the same time.

We also observed an increase in *Bifidobacterium* abundance in the group treated with coconut oil (Figure 5). A high bacterial diversity in pigs’ rectal swabs, together with an increased abundance of probiotic bacteria such as *Lactobacillus* and *Bifidobacterium*, is positive and desirable in pig breeding. Contrary to the results of Puyalto et al. [12], we observed an insignificant increase in *Enterobacteriaceae* during the experiment in the group with the coconut oil treatment, without a negative effect on the health of the pigs. On the other hand, the abundance of *Corynebacterium* in the pig group treated with lauric acid in the form of coconut oil was significantly lower in comparison with that in the animals fed a normal diet.

Megahed et al. [40] listed *Psychrobacter* and *Corynebacterium* among unfavorable taxa in the GITs of growing pigs. Thus, a positive effect of coconut oil treatment is manifested by a lower abundance of *Corynebacterium* and a significant reduction in *Psychrobacter* genera in pigs’ rectums after treatment with feed containing coconut oil.

Homogeneity within each experimental group is crucial for the determination of the feed supplementation effect. However, it is difficult to ensure the homogeneity of pig microbiomes, as they change within a few days, especially after weaning [41]. In our experiment, we found several significant differences between groups before treatment (*Bacteroidetes* and *Firmicutes*) despite pigs being randomized before the start of the experiment. Prominent inter-individual variation has also been reported in many other studies with pigs [32,42]. Inter-individual microbiome variation is common and was detected in animals as well as humans. An analysis of the gut microbiota in a probiotic-fed fish population and the fecal microbiota of horses showed that bacterial populations are influenced by day-to-day fluctuations and inter-individual differences [43,44]. Substantially increased numbers of individuals are required to create complex images of microbiome changes that are dependent on feed. Nevertheless, we observed differences between groups that indicated a reduction in unfavorable groups of microorganisms, while groups such as *Lactobacillus* and *Bifidobacterium* increased in response to the addition of coconut oil to feed. According to Gueimonde et al. [45], such bacteria play an important role in the prevention of gut colonization by pathogens. This benefit is provided by a competitive “fight” for nutrients and by the production of proteins that are capable of bacteriolysis. Stabilization or improvement of microbiota during the growth of pigs has positive effects on their performance and on the prevention of some diseases, especially diarrhea caused by virulent *E. coli* strains, *Lawsonia*, or *Salmonella* [46]. According to other authors [47,48,49], medium-chain fatty acids, including lauric acid and its ester derivatives, may inhibit the growth of *Salmonella* and *Brachyspira*. These authors also claimed that medium-chain fatty acids could be used as antibiotics replacements.

## 5. Conclusions

In the rectal swab samples of investigated pigs, the most abundant phyla were *Firmicutes* and *Bacteroidetes*, while *Megasphaera* and *Prevotella* were the most abundant genera. During the experiment, microbiomes shifted toward a higher abundance of *Firmicutes* and a lower abundance of *Bacteroidetes*. Despite some differences in the microbiomes of pigs before treatment, we were able to prove that coconut oil addition mainly had a positive effect, which is probably connected to elevated levels of lauric acid. The addition of coconut oil increased the abundance of beneficial microorganisms such as *Lactobacillus* or *Bifidobacterium* while decreasing some unfavorable taxa. Positive shifts in the microbiome indicate that coconut oil has selective antimicrobial effects and can improve pig health, reduce the use of antibiotics, and result in higher pig performance. The exact amount of coconut oil that should be added to feed and its combination with other supplements need to be determined in further research.

## Figures and Tables

**Table 1:** Composition, nutritional parameters, and proportion of fatty acids in feed rations provided during the experiment.

Pigs Group	Normal	Oil Supplement
	Composition of feed ration (%)	
Commercial feed mixture	100	99.70
Commercial coconut oil	-	0.30
	Nutritional parameters (%)	
Dry matter	89.30	89.10
Crude protein	15.90	16.27
Lysine	1.16	1.15
Crude fat	3.33	4.04
Crude fiber	4.23	4.76
Nitrogen-free extract	61.85	60.20
Starch	47.20	43.90
Sugar	2.98	2.40
Ash	3.99	3.83
MEpigs (MJ.kg^−1^)	15.70	15.80
Proportion of fatty acids (g.100 g^−1^ Fatty acids)		
C8:0	-	0.73
C10:0	-	0.64
C12:0	0.25	5.34
C14:0	0.67	2.49
C16:0	18.73	17.68
C16:1	1.72	1.60
C17:0	0.21	0.19
C18:0	5.67	5.47
C18:1cis n9	27.81	25.80
C18:2cis n6	38.35	34.09
C18:3 n3	3.34	2.94
C20:0	0.20	0.19
C20:1 n9	0.48	0.44
C20:2 n6	0.18	0.16
C20:4 n6	0.13	0.12
C22:0	0.14	0.12
Σ unidentified	2.12	2.00
PUFA	42.00	37.31
MUFA	30.01	27.84
SFA	25.87	32.85
Σn3/Σn6	0.09	0.09
Σn6/Σn3	11.57	11.69

MEpigs—metabolizable energy for pigs in megajoules per kilogram; PUFA—polyunsaturated fatty acids; MUFA—monounsaturated fatty acids; SFA—saturated fatty acids; Σ3—sum of omega-3 fatty acids; Σ6—sum of omega-6 fatty acids.

**Table 2:** Microbial community members with occurrences of at least 0.5% in rectal swabs of pigs fed a normal diet and a diet supplemented with coconut oil and the significance of changes between groups during feeding.

Taxonomic Rank						Relative Abundance (%)				*p*-Value			
Phyllum	Class	Order	Family	Genus	OTU	Normal diet Before treatment	Normal diet After treatment	Oil supp. group Before treatment	Oil supp. group After treatment	In-time change in Normal diet group	In-time change in Oil supp. group	Difference Normal-Oil Before treatment	Difference Normal-Oil After treatment
*Actinobacteria*						0.87	2.60	1.33	1.85	0.020	0.232	0.315	0.356
	*Actinobacteria*					0.87	2.60	1.33	1.85	0.020	0.232	0.315	0.356
		*Actinomycetales*				0.42	0.79	0.52	0.21	0.098	0.193	0.842	0.006
			*Corynebacteriaceae*			0.41	0.77	0.51	0.20	0.098	0.160	0.780	0.006
				*Corynebacterium*		0.41	0.77	0.51	0.20	0.098	0.160	0.780	0.006
		*Coriobacteriales*				0.45	1.79	0.79	1.42	0.004	0.002	0.315	0.780
			*Coriobacteriaceae*			0.45	1.79	0.79	1.42	0.004	0.002	0.315	0.780
				*Enterorhabdus*		0.03	0.36	0.12	0.53	0.012	0.004	0.010	0.211
					OTU053	0.03	0.36	0.12	0.52	0.012	0.006	0.006	0.243
				*Olsenella*		0.31	1.15	0.52	0.45	0.203	0.695	0.842	0.211
					OTU067	0.05	0.61	0.05	0.07	0.039	0.944	0.773	0.015
*Bacteroidetes*						49.53	30.31	34.96	29.49	0.008	0.492	0.013	0.905
	*Bacteroidia*					49.46	30.28	34.96	29.48	0.008	0.492	0.013	0.905
		*Bacteroidales*				49.46	30.28	34.96	29.48	0.008	0.492	0.013	0.905
			*Porphyromonadaceae*			0.42	1.13	0.49	1.10	0.020	0.037	0.905	0.905
				*Barnesiella*		0.35	0.93	0.38	0.90	0.098	0.037	0.842	0.905
					OTU022	0.29	0.85	0.29	0.60	0.098	0.049	0.720	0.604
			*Prevotellaceae*			48.98	29.00	34.44	28.22	0.008	0.432	0.017	1.000
				*Alloprevotella*		1.14	0.18	0.35	1.04	0.012	0.037	0.182	0.001
					OTU033	1.01	0.12	0.16	0.34	0.012	0.131	0.030	0.133
					OTU066	0.05	0.05	0.09	0.59	0.834	0.084	0.189	0.008
				*Prevotella*		47.80	28.77	34.05	27.12	0.008	0.322	0.017	0.842
					OTU002	12.16	3.29	8.62	5.11	0.004	0.275	0.243	0.315
					OTU003	7.54	6.48	5.52	6.23	0.496	0.922	0.113	0.780
					OTU004	4.25	3.74	3.38	2.47	0.910	0.432	0.243	0.278
					OTU006	3.31	2.10	2.66	2.61	0.164	0.625	0.400	0.842
					OTU008	3.06	0.44	2.40	0.03	0.129	0.002	0.968	0.013
					OTU009	2.29	1.15	0.78	1.24	0.652	0.432	0.004	0.905
					OTU010	1.08	2.81	0.68	0.61	0.129	0.922	0.133	0.053
					OTU012	1.73	0.64	1.32	1.18	0.027	1.000	0.278	0.028
					OTU015	0.98	0.80	1.00	1.10	0.496	0.846	0.720	0.604
					OTU016	0.88	1.19	1.13	0.34	0.426	0.557	0.968	0.065
					OTU017	2.67	0.01	0.58	0.05	0.107	0.193	0.837	0.263
					OTU023	0.80	0.61	0.54	0.34	0.820	0.906	0.487	0.243
					OTU024	0.73	0.67	0.48	0.33	0.820	0.322	0.079	0.079
					OTU026	0.77	0.26	0.36	0.40	0.055	1.000	0.053	0.780
					OTU027	1.11	0.12	0.40	0.15	0.008	0.049	0.053	0.967
					OTU030	0.55	0.25	0.41	0.38	0.004	0.625	0.356	0.356
					OTU031	0.31	0.72	0.06	0.23	0.496	0.084	0.165	0.356
					OTU037	0.06	0.07	0.10	0.81	0.353	0.037	0.539	0.006
					OTU039	0.52	0.09	0.22	0.12	0.008	0.160	0.156	0.400
					OTU041	0.25	0.54	0.12	0.02	0.834	0.294	0.838	0.072
					OTU045	0.00	0.00	0.35	0.58	0.201	0.813	0.007	0.256
*Firmicutes*						48.86	64.46	62.71	66.03	0.027	0.625	0.013	0.780
	*Bacilli*					1.61	2.55	1.22	3.17	0.098	0.064	0.661	0.780
		*Lactobacillales*				1.61	2.55	1.22	3.17	0.098	0.064	0.661	0.780
			*Lactobacillaceae*			1.60	2.52	1.16	3.10	0.098	0.049	0.720	0.604
				*Lactobacillus*		1.60	2.52	1.16	3.10	0.098	0.049	0.720	0.604
					OTU013	0.71	0.75	0.63	1.58	0.734	0.027	0.968	0.035
					OTU014	0.44	1.03	0.21	1.28	0.129	0.432	0.905	0.004
					OTU025	0.43	0.70	0.26	0.15	0.129	0.105	0.720	0.010
	*Clostridia*					1.16	1.57	2.29	1.94	0.570	0.846	0.497	0.497
		*Clostridiales*				1.16	1.57	2.29	1.94	0.570	0.846	0.497	0.497
			*Lachnospiraceae*			0.52	0.71	0.88	0.51	0.496	0.131	0.400	0.604
			*Ruminococcaceae*			0.49	0.67	1.00	0.98	0.250	0.922	0.315	0.133
	*Negativicutes*					46.00	60.15	59.03	60.85	0.039	0.922	0.053	0.968
		*Selenomonadales*				46.00	60.15	59.03	60.85	0.039	0.922	0.053	0.968
			*Acidaminococcaceae*			0.38	0.59	1.21	1.33	0.164	0.846	0.278	0.356
				*Phascolarctobacterium*		0.21	0.28	0.86	1.20	0.496	0.695	0.356	0.113
					OTU021	0.21	0.28	0.86	1.20	0.496	0.695	0.356	0.113
			*Veillonellaceae*			45.62	59.56	57.83	59.51	0.039	0.922	0.053	0.968
				*Dialister*		1.85	3.34	2.62	1.99	0.203	0.432	0.780	0.243
					OTU005	1.79	3.21	2.48	1.83	0.203	0.432	0.842	0.211
				*Megasphaera*		37.27	52.80	50.08	55.24	0.027	0.375	0.182	0.780
					OTU001	35.28	50.88	47.74	53.03	0.027	0.322	0.182	0.780
					OTU020	0.54	0.26	0.65	0.59	0.098	0.492	0.842	0.156
				*Mitsuokella*		1.78	1.93	1.26	0.62	0.820	0.004	0.095	0.028
					OTU018	0.88	0.59	0.49	0.13	0.129	0.002	0.079	0.095
					OTU019	0.60	0.66	0.46	0.29	0.910	0.037	0.243	0.035
					OTU029	0.18	0.62	0.13	0.17	0.250	0.846	0.278	0.013
				*Propionispira*		1.58	0.45	1.39	0.86	0.020	0.131	1.000	0.156
					OTU007	1.57	0.45	1.38	0.85	0.020	0.131	1.000	0.156
				*Selenomonas*		2.96	0.95	2.26	0.53	0.004	0.010	0.315	0.243
					OTU011	2.33	0.42	1.54	0.30	0.004	0.027	0.243	0.447
*Proteobacteria*						0.60	2.21	0.82	2.42	0.004	0.275	0.447	0.211
	*Deltaproteobacteria*					0.14	0.63	0.43	0.90	0.020	0.193	0.133	0.549
		*Bdellovibrionales*				0.12	0.43	0.41	0.84	0.055	0.275	0.035	0.497
			*Bdellovibrionaceae*			0.12	0.43	0.41	0.84	0.055	0.275	0.035	0.497
				*Vampirovibrio*		0.12	0.43	0.41	0.84	0.055	0.275	0.035	0.497
	*Epsilonproteobacteria*					0.05	0.66	0.10	0.44	0.012	0.275	0.437	0.182
		*Campylobacterales*				0.05	0.66	0.10	0.44	0.012	0.275	0.437	0.182
			*Campylobacteraceae*			0.05	0.66	0.10	0.41	0.012	0.275	0.437	0.182
				*Campylobacter*		0.05	0.66	0.10	0.41	0.012	0.275	0.437	0.182
					OTU050	0.05	0.52	0.10	0.41	0.164	0.275	0.437	1.000
	*Gammaproteobacteria*					0.40	0.91	0.28	1.07	0.055	0.922	0.549	0.079
		*Enterobacteriales*				0.09	0.21	0.02	0.74	0.426	1.000	0.966	0.066
			*Enterobacteriaceae*			0.09	0.21	0.02	0.74	0.426	1.000	0.966	0.066
				*Escherichia/Shigella*		0.00	0.12	0.02	0.74	0.036	0.726	0.131	0.537
					OTU028	0.00	0.12	0.02	0.74	0.036	0.726	0.131	0.537
	*Gammaproteobacteria*					0.40	0.91	0.28	1.07	0.055	0.922	0.549	0.079
		*Pseudomonadales*				0.20	0.50	0.12	0.02	0.426	0.037	0.182	0.003
			*Moraxellaceae*			0.20	0.50	0.12	0.02	0.426	0.037	0.182	0.003
				*Psychrobacter*		0.20	0.50	0.12	0.02	0.426	0.027	0.182	0.002
					OTU043	0.20	0.50	0.12	0.02	0.426	0.027	0.182	0.002
